# Simultaneous Quantification of the Acetylome and Succinylome by ‘One‐Pot’ Affinity Enrichment

**DOI:** 10.1002/pmic.201800123

**Published:** 2018-08-19

**Authors:** Nathan Basisty, Jesse G Meyer, Lei Wei, Bradford W Gibson, Birgit Schilling

**Affiliations:** ^1^ The Buck Institute for Research on Aging 94945 Novato CA USA; ^2^ Amgen 94080 South San Francisco CA USA

**Keywords:** acetylation, data‐independent acquisition, immunoaffinity enrichment, posttranslational modification, succinylation

## Abstract

Protein posttranslational modifications (PTMs) are of increasing interest in biomedical research, yet studies rarely examine more than one PTM. One barrier to multi‐PTM studies is the time cost for both sample preparation and data acquisition, which scale linearly with the number of modifications. The most prohibitive requirement is often the need for large amounts of sample, which must be increased proportionally with the number of PTM enrichment steps. Here, a streamlined, quantitative label‐free proteomic workflow—“one‐pot” PTM enrichment—that enables comprehensive identification and quantification of peptides containing acetylated and succinylated lysine residues from a single sample containing as little as 1 mg mitochondria protein is described. Coupled with a label‐free, data‐independent acquisition (DIA), 2235 acetylated and 2173 succinylated peptides with the one‐pot method are identified and quantified and peak areas are shown to be highly correlated between the one‐pot and traditional single‐PTM enrichments. The ‘one‐pot’ method makes possible detection of multiple PTMs occurring on the same peptide, and it is shown that it can be used to make unique biological insights into PTM crosstalk. Compared to single‐PTM enrichments, the one‐pot workflow has equivalent reproducibility and enables direct assessment of PTM crosstalk from biological samples in less time from less tissue.

Posttranslational modifications (PTMs) dynamically regulate a diverse set of protein functions, including turnover,^1^ binding,^2^ signaling,^3^, ^4^ localization,^5^ and interaction with other cellular molecules.^2^, ^3^ There has been an ‘explosion’ in the number of studies using antibody‐based affinity enrichment of PTM containing peptides to quantify modifications, but rarely do these studies consider multiple modifications in parallel. Still, several examples of multi‐PTM crosstalk have been presented.^6^, ^7^, ^8^, ^9^ One barrier to these multi‐PTM studies is the time cost for both sample preparation and instrument data acquisition, which typically scale linearly with the number of modifications studied. Additionally, PTM studies typically require large amounts of input sample protein, which increases with the number of PTMs investigated in traditional enrichment workflows. There is a critical need for more efficient, multi‐PTM quantification methods, without which progress toward complete understanding of crosstalk between distinct PTM signals will remain stagnant. While the importance to investigate PTM crosstalk between different modifications is well acknowledged,^10^ practical tools to efficiently implement experimental workflows can still be optimized. Current approaches^10^ include i) parallel enrichments of different PTMs, ii) serial PTM enrichments, and iii) intact protein analysis. One pot enrichment workflows will address challenges, such as restriction in sample amount (small amounts of input material), and be advantageous because of reduction in sample preparation time, reduction in MS instrument acquisition time, less sample handling (e.g., one pot vs serial), etc. Toward the long‐term goal of comprehensive PTM studies, here we demonstrate that simultaneous, one‐pot enrichment of peptides containing acetyl‐lysine and succinyl‐lysine coupled with label‐free data‐independent acquisition (DIA) quantification can achieve comprehensive and reproducible profiling of PTMs that is equivalent to independent PTM enrichments. We show directly that one‐pot enrichment is quantitatively equivalent compared to individual enrichment steps, as well as previously described serial immunoaffinity enrichment methods.^9^, ^11^

We further show that this strategy can be used to detect peptides co‐modified by both acetylation and succinylation, which opens unique opportunities to study PTM crosstalk. This workflow requires no isotopic labeling reagents, only 1 mg protein per sample, and reduces the time required for sample preparation, instrument data acquisition, and data analysis by half. Therefore, proteomic quantification of multiple PTMs by this one‐pot enrichment method makes multi‐PTM profiling a feasible, cost‐effective approach for biological studies.

The one‐pot workflow implements the simultaneous enrichment of multiple PTMs using a combination of antibodies against acetyl‐lysine and succinyl‐lysine in the same tube (Figure 1A, PTM scan kits #13416 and #13764, Cell Signaling Technology, Danvers, MA). In contrast, serial enrichment uses unbound peptides from a single‐PTM enrichment for subsequent enrichment with a second antibody (Figure S1, Supporting Information).^9^, ^11^ We compared the quantitative performance of each strategy: single‐PTM, serial‐PTM, or one‐pot‐PTM enrichment, using trypsin‐digested peptides from 1 mg of mouse liver mitochondrial protein and DIA analysis as previously described.^12^ Compared with single‐ or serial‐PTM enrichments, one‐pot enrichment reduces the number of samples by half for all steps after enrichment, therefore significantly reducing costs and time required to perform downstream processing steps such as desalting, as well as data acquisition, processing, and analysis (Figure 1B).

**Figure 1:**
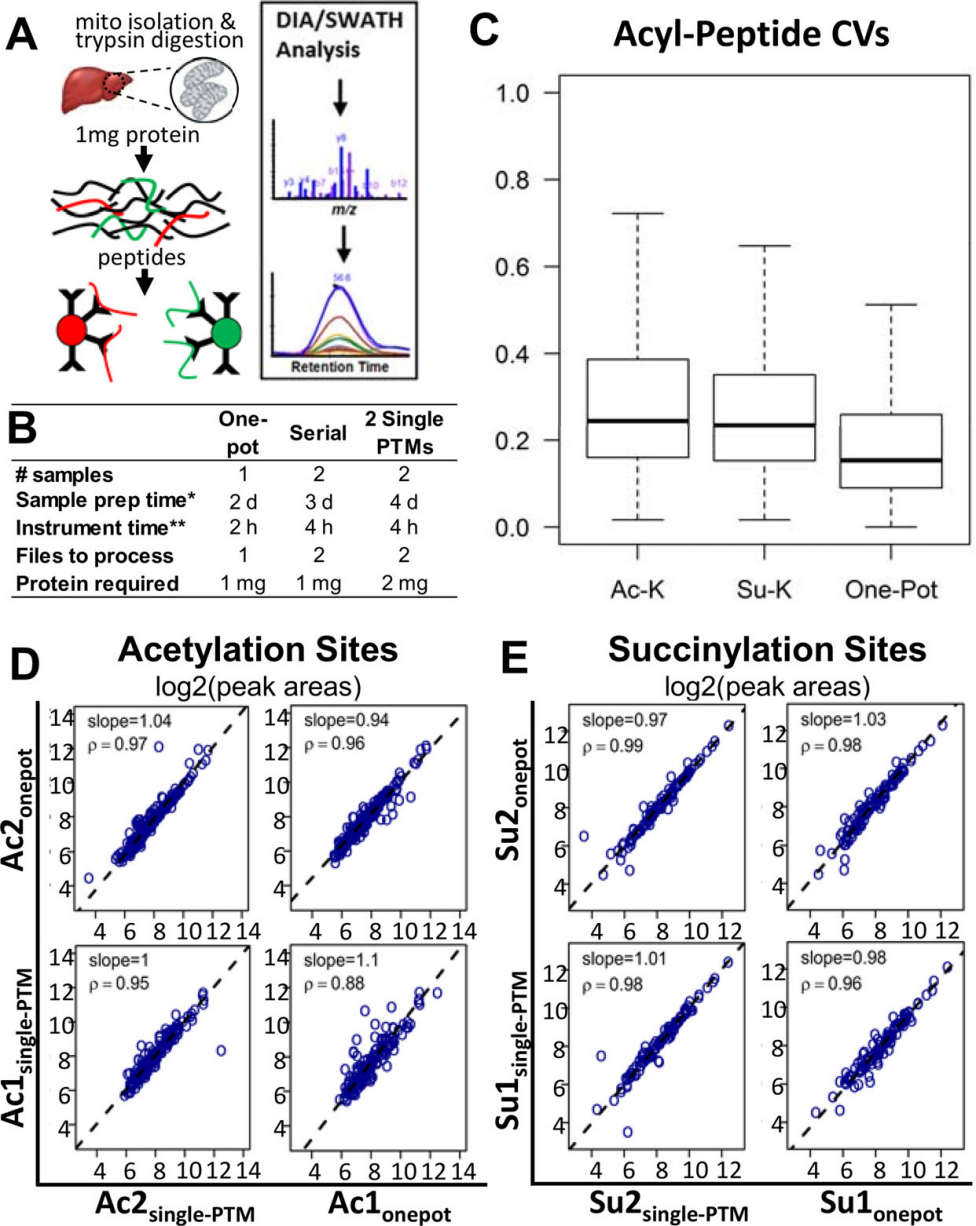
Workflow and reproducibility of one‐pot enrichment of PTM's. A) Antibodies are combined into a single tube and immunoaffinity enrichment of both modifications is performed in a single step, followed by DIA analysis. B) Table summary of time and resource requirements for one‐pot, serial, and single pulldowns. C) Boxplot of coefficients of variation of all acetylated or succinylated peptides identified following acetyl‐lysine, succinyl‐lysine, or one‐pot enrichments. The median coefficient of variation (CV) for modified peptide areas was 15.3%, 24.5%, and 23.4% for one‐pot, acetyl‐lysine, and succinyl‐lysine affinity enrichments, respectively. D) Correlation of acetylation site‐level quantification of two one‐pot pulldowns performed in parallel with two acetylation‐only pulldowns (*d, days; **h, hours). E) Correlation of succinylation site‐level quantification one‐pot enrichments to succinylation‐only pulldowns. Ac, acetylated; Su, succinylated. Ac_single‐PTM_, acetyl‐lysine pulldown sites; Ac_onepot_, acetylation sites from one‐pot pulldown; Su_single‐PTM_, succinyl‐lysine pulldown sites; Su_onepot_, succinylation sites from one‐pot pulldown.

To compare each strategy (i.e., single‐PTM, serial‐PTM, or one‐pot‐PTM enrichment), we prepared identical aliquots of peptides from trypsin digestions of 1 mg of mouse liver mitochondrial protein and performed parallel enrichments with each strategy (see also detailed Supporting Information Methods). Enriched peptides from duplicate or quadruplicate process replicates of each strategy were analyzed by DIA on a TripleTOF 5600 mass spectrometer (AB Sciex, Concord, Canada) with 64 variable‐size precursor isolation windows covering *m/z* 400–1250,^12^ and acylated peptides were quantified with Spectronaut (v. 10, Biognosys, MA, USA).^13^ Spectral libraries were generated by DDA analysis of duplicate one‐pot enrichments (1 mg of protein each) or parallel acetyl‐lysine, succinyl‐lysine, and one‐pot enrichments in duplicate (1 mg protein each). Raw data is available at MassIVE (MSV00081906) and ProteomeXchange (PXD008640), and spectral libraries are available at PanoramaWeb (https://panoramaweb.org/project/Schilling/OnePot_Basisty/begin.view?).

Quantitative performance and reproducibility of the one‐pot enrichment method was assessed by calculating coefficients of variation and correlation of peak areas between technical process replicates of one‐pot and single‐PTM enrichments (Figure 1C–E; Tables S1 and S2, Supporting Information). The median coefficient of variation (CV) for modified peptide areas was 15.3%, 24.5%, and 23.4% for one‐pot, acetyl‐lysine, and succinyl‐lysine affinity enrichments, respectively. Figure S2, Supporting Information provides additional CV displays, such as a CV density plot for one‐pot versus individual affinity enrichments. PTM site‐level correlation between single‐PTM and one‐pot PTM enrichments were indistinguishable for both acetylation and succinylation sites, as were peptide‐level and fragment‐level areas (Figures S3–S5, Supporting Information). Similar quality of correlation was found between one‐pot and serial‐PTM enrichments at the site‐level, peptide‐level, and fragment‐level (Figures S5–S7 and Table S5, Supporting Information). Together, the strong correlation between single‐PTM, serial‐PTM, and one‐pot PTM enrichments establish their quantitative equivalence.

A notable advantage of the ‘one‐pot’ workflow is the capability to detect peptides containing both an acetylated and a succinylated lysine residue. Although serial enrichment methods^9^, ^11^ are capable of separately enriching and analyzing multiple PTMs from a single sample, the one‐pot enrichment is the first method to enable the analysis of multiple enriched PTMs simultaneously and comprehensively. Database searching for two PTMs at once, rather than in separate searches, however, creates a larger search space and may result in a larger overlap between target and decoy peptide scores and thus decreased identification sensitivity.^13^ We therefore compared the number of modified peptides identified from combined and individual PTM database searches with the same data (Figure 2A; Figure S9 and Table S3, Supporting Information). Performing separate searches yielded acylated peptide IDs similar in number to individual searches (±7–10%) and most peptides identified by either individual or combined searches overlapped. Using the combined search, we identified 2235 acetylated and 2173 succinylated peptides, totaling over 4200 acylation sites from each sample derived from 1 mg of starting protein. Notably, this combined search of peptides from one‐pot enrichment allowed detection of multiple different PTMs on the same peptide. An example of a peptide sequence found with two succinyl modifications or one succinyl and one acetyl modification is shown in Figure 2B and 2C and Figures S10 and S11, Supporting Information. The DIA approach also provides a powerful advantage for detecting and quantifying peptides of identical *m/z* values with distinct and different sites of lysine modification, which is not possible at the MS1 precursor‐ion level. In these cases, the site‐localized DIA MS2 product ions can differentiate the extracted chromatographic peaks of precursor ions with otherwise identical sets of product ions (Figure S12, Supporting Information). These PTM site‐localization or PTM site‐differentiating product ions can subsequently be used for quantification even if PTM‐isomers were to co‐elute.

**Figure 2:**
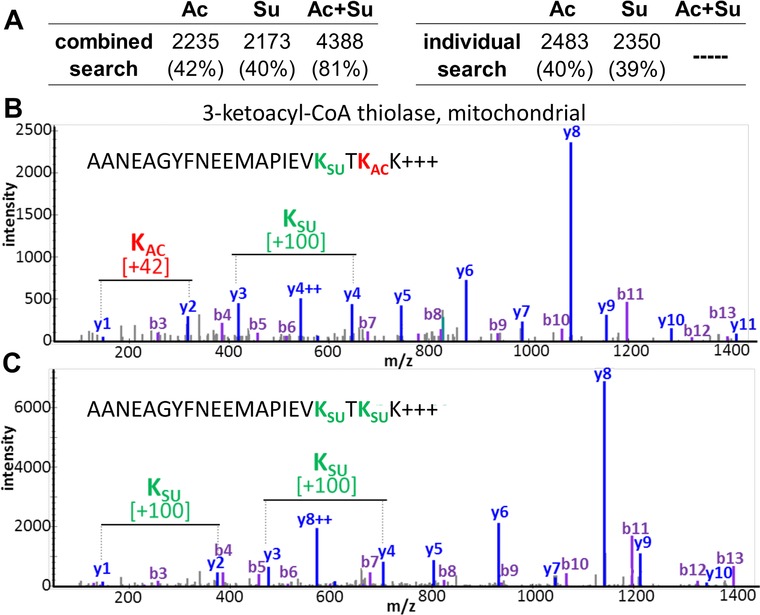
Database searching for acetylation and succinylation together versus individually. A) Table summary of the total number of acetylated and succinylated peptides (and percentage of total peptide IDs containing either modification) identified from performing database searching of both acyl modifications combined or individually. B) MS/MS spectrum of a peptide containing both acetylation and succinylation on K209 and K211, respectively, on the protein 3‐ketoacyl‐CoA thiolase, mitochondrial (precursor ion selected at *m/z* 828.07). C) MS/MS spectrum of the same peptide succinylated on both corresponding lysines (precursor ion selected at *m/z* 847.40). Ac, acetylated; Su, succinylated. Peptide MS/MS spectra were taken from a DDA spectral library.

To investigate crosstalk between lysine acetylation and succinylation, we determined which sites were exclusively either acetylated or succinylated as well as indiscriminately modified sites, i.e., lysines that are modified by either (Figure 3A; Table S4, Supporting Information). Strikingly, over 40% of acetylation and succinylation sites were identical, suggesting high potential for crosstalk between these modifications. Ontology term enrichment analysis, performed with the ConsensusPathDB tool,^14^ revealed that several protein complexes—ATP synthase, pyruvate dehydrogenase complex (PDH), and branched‐chain α‐ketoacid dehydrogenase complex (BCKDH)—were exclusively enriched with indiscriminately modified acylation sites (Figure 3B). The largest complex, ATP synthase, was enriched with the same identical acylation sites in 13 of 15 subunits, lacking acylation sites in only the two subunits integral to the proton channel (Figure 3C). The enrichment of dual acetylation and succinylation sites among these protein complexes suggests that crosstalk may serve a role in regulating their function, or perhaps help orchestrate their assembly. Further experimentation is needed to determine the role of crosstalk in this complex.

**Figure 3:**
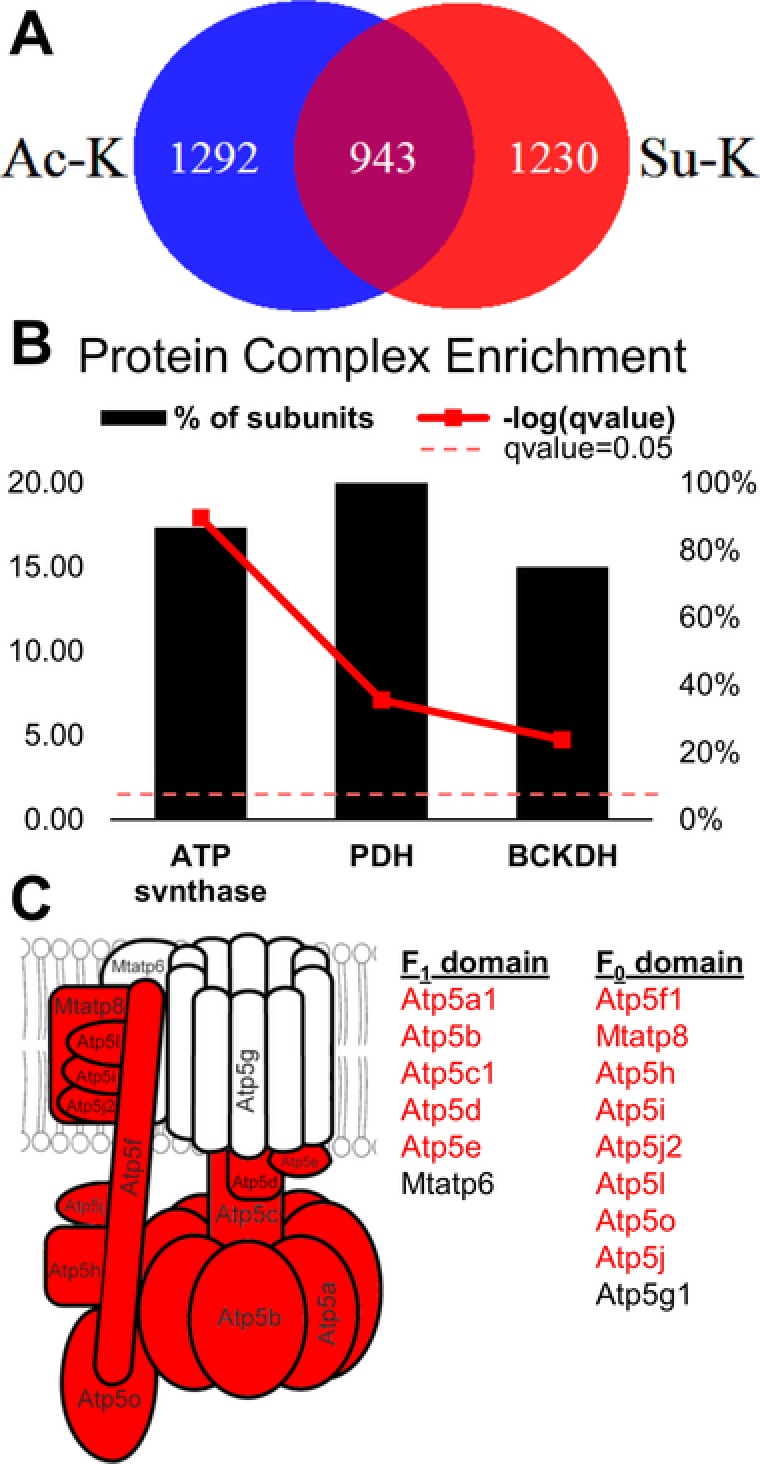
Crosstalk between lysine acetylation and succinylation. A) Venn diagram of acetylation and succinylation sites identified following one‐pot enrichment. B) Term enrichment analysis of protein complexes among indiscriminately modified acylation sites (sites that are both acetylated and succinylated). Black bars represent the percent of total subunits containing sites. The red line represents the significance of enrichment by Fischer exact test, and the dotted line is the significance threshold. C) Schematic of ATP synthase highlighting the subunits containing indiscriminately modified acylation sites in red. Ac, acetylated; Su, succinylated.

The ability for biomedical researchers to perform comprehensive and quantitative PTM profiling as a component of a biological study is limited by several factors, including protein quantity required for each PTM enrichment and the time required for sample preparation, MS data acquisition, and data analysis. By cutting each of these requirements in half, the one‐pot enrichment method enables researchers to perform robust and reproducible PTM profiling studies using protein from a portion of a tissue or biofluid while sparing the remainder of the sample for other experimental approaches. Furthermore, by enriching for several PTMs at the same time and from the same sample, the one‐pot workflow opens an opportunity to study PTM crosstalk. Although, here we demonstrate simultaneous acetyl‐ and succinyl‐ lysine enrichment, the method can presumably be extended to a near‐arbitrary number of PTMs. Additional studies are needed to assess the limit of how many simultaneous PTM enrichments are possible as some affinity enrichment reagents may be incompatible. In conclusion, the one‐pot PTM enrichment method described here offers multiple benefits for multi‐PTM studies and enables efficient and cost‐effective method for examining PTM crosstalk.

## Conflict of Interest

The authors declare no conflict of interest.

## Supporting information

Supporting information.

Supporting information.

Supporting information.

Supporting information.

Supporting information.

Supporting information.

Supporting information.

Supporting information.

Supporting information.
