# Supplementary material for: Simultaneous Quantification of the Acetylome and Succinylome by ‘One‐Pot’ Affinity Enrichment
Source: Proteomics. 2018 Aug 19;18(17):1800123. doi: 10.1002/pmic.201800123 (PMC6175148; doi:10.1002/pmic.201800123)
Supplement: Supplementary file 1 — Supporting information. [file PMIC-18-na-s001.docx]

**One-pot (acetyl & succinyl) peptide affinity enrichment protocol:**

**Materials:**

- [PTMScan® Acetyl-Lysine Motif [Ac-K] Kit](https://www.cellsignal.com/products/proteomic-analysis-products/acetyl-lysine-motif-ac-k-kit/13416), Cell Signaling Technology (CST) #13416
- [PTMScan® Succinyl-Lysine Motif [Succ-K] Kit](https://www.cellsignal.com/products/proteomic-analysis-products/succinyl-lysine-motif-succ-k-kit/13764?N=4294956287&Ntt=succinyl+kit&fromPage=plp&_requestid=66372), Cell Signaling Technology (CST) #13764
- [Corning® 1-200µL Flat 0.2mm Thick Gel-Loading Pipet Tips, Natural, Nonsterile, 200 Tips/Rack, 2 Racks/Case, 400 Tips/Case](http://catalog2.corning.com/LifeSciences/en-US/Shopping/ProductDetails.aspx?productid=4884(Lifesciences)), Product #4884
- [Empore™ Solid Phase Extraction Disks](http://www.sigmaaldrich.com/catalog/product/supelco/66883u?lang=en&region=US), C18, 47mm, Sigma #6883-U
- [Burdick and Jackson water](http://www.buyhoneywellbandj.com/Water-for-HPLC-4liter-Case-of/M/B007PJJMBM.htm), Honeywell #AH365-4
- [Triethylammonium bicarbonate buffer 1.0 M](http://www.sigmaaldrich.com/catalog/product/sigma/t7408?lang=en&region=US), pH 8.5±0.1, Sigma #**T7408**
- [Formic Acid](http://www.sigmaaldrich.com/catalog/product/fluka/94318?lang=en&region=US)**, Sigma #94318-50ML-F**
- [Pierce BCA protein assay kit](https://www.thermofisher.com/order/catalog/product/23225) #23225
- [Oasis HLB 1 cc Vac Cartridge, 30 mg Sorbent per Cartridge, 30 µm Particle Size, 1000/pk](http://www.waters.com/waters/partDetail.htm?locale=en_US&partNumber=186003908), Waters #186003908
- [Trypsin](https://www.promega.com/products/mass-spectrometry/proteases-and-surfactants/trypsin-for-protein-characterization/trypsin-reagents/sequencing-grade-modified-trypsin_-frozen/?activeTab=0), Product #V5113, Promega
- A peptide standard for spike in, e.g., the indexed retention time (iRT) or [HRM Calibration Kit](https://biognosys.com/shop/hrm-calibration-kit) from Biognosys.
- Acetonitrile, Product #015-4, Honeywell Burdick and Jackson

*** Additional material needed for the solutions see below. Further materials are listed under the day they are used***

**Solutions:**

***kept cold throughout when using***

- Burdick and Jackson Water
- 1x D-PBS (10X solution in mini fridge)
- IAP buffer (10X aliquots in -20 ºC freezer)
  - 50 mM 3-(*N*-morpholino)propanesulfonic acid **(**MOPS)/NaOH pH 7.2
  - 10 mM Na_2_HPO4
  - 50 mM NaCl

**Day 0 - Preparation of tissue**

**Affinity Lysis Buffer:**

- 50 mM Triethylammonium bicarbonate (TEAB)
- 20 mM nicotinamide (deacetylase inhibitor)
- [1M trichostatin A](http://www.sigmaaldrich.com/catalog/product/sigma/t8552?lang=en&region=US) (TSA), Product #T8552, SIGMA (deacetylase inhibitor)
- protease inhibitor cocktail

Isolate mitochondrial factions from tissues or prepare total tissue lysates, and resuspend in affinity lysis buffer.

*** On the TripleTOF 5600 the final concentration of sample injected for MS acquisition is ideally ~1 µg of protein in 2-5 µL of total volume ***

**Day 1 – BCA & Trypsin digestion (2-3 hours, and overnight digestion):**

**Materials:**

- BCA Kit
- 50 mM TEAB
- 8 M [Urea](https://www.thermofisher.com/order/catalog/product/29700), product #29700 Thermo
- 1 M Dithiothreitol ([DTT](http://www.sigmaaldrich.com/catalog/product/sial/d0632?lang=en&region=US)), product #D0632, SIGMA
- 10 M Iodoacetamide ([IAA](http://www.sigmaaldrich.com/catalog/product/sigma/i6125?lang=en&region=US)), product #I6125, SIGMA
- Trypsin

1. Perform BCA assay on dilutions of mitochondrial protein to determine protein concentration, and aliquot 1 mg of total protein for affinity enrichment.
   1. Note: BCA is only compatible with urea up to 3 M
2. For each sample, add:
   1. 100 µL 8 M urea,
   2. 10 µL 1M TEAB pH 8.5,
   3. 10 µL 10% maltoside
   4. remaining volume of water (final volume 200 µL) to mitochondria and
   5. Vortex to lyse.
   6. Add 1M DTT to final concentration of 4.5 mM to reduce proteins
      1. Incubate 30 minutes at 37 ºC with shaking.
      2. Let samples cool to room temperature.
   7. Add Iodoacetamide (IAA) to a final concentration of 10 mM
      1. Incubate 30 minutes in the dark.
   8. Dilute samples with 50 mM TEAB to 980 µL
   9. Add 20 µg of trypsin for a final volume of 1 mL.
      1. Incubate for 2 hours or O/N

**Day 2 – HLB clean-up and Antibody-bead affinity enrichment (6-8 hours):**

**Materials:**

- CST Acetyl Antibody
- CST Succinyl Antibody
- Waters OASIS HLB sample cleanup materials
- 10% Formic Acid
- 80% ACN/0.2% FA/19.8% Water
- 0.2% FA in water
- IAP buffer
- D-PBS
- Trypsin

1. Add 100 µL 10% formic acid to samples to quench digests and precipitate lipids.
2. Spin at 1,800 rcf for 15 minutes at room temperature to pellet insoluble material.
3. Desalt peptides with Waters HLB SPE cartridges:
   1. **Wet** cartridge 2X with 0.8 mL of 80% ACN/0.2% FA/19.8% Water
   2. **Equilibrate** cartridge 3X with 0.8 mL 0.2% FA in water
   3. **Load** peptides
   4. **Wash** peptides 3x with 0.8 mL 0.2% FA in water
   5. **Elute** peptides 1X with 0.8 mL 80% ACN/0.2% FA/19.8% Water and 1X with 0.4 mL 80% ACN/0.2% FA/19.8% Water.
   6. **Dry** completely in SpeedVac (about 2-3 hours).
4. Re-suspend peptides from 1 mg digest desalted by HLB with 1.4 mL cold IAP buffer. Pipette and vortex to mix (ensure pH is around 7).
5. Centrifuge 10 minutes at 10,000 rcf in cold room. A small pellet may appear.

****Preparing the antibody-beads prior to incubation with peptides****

1. Add 1 mL cold 1X PBS to a tube of CST antibody. Mix and transfer suspension to a new 1.5 mL microfuge tube and spin down with mini microfuge 30 seconds at room temperature. Remove / aspirate most of the PBS.
2. Wash with 1 mL cold 1X PBS. Spin in mini microfuge 30 seconds at room temp. Remove/Aspirate PBS.
3. Repeat step 4 twice more for a total of **four, 1mL PBS washes.**
4. Re-suspend beads in PBS. For individual Kac or Ksu enrichment: Use ¼ of the beads from one PTM Scan Tube for each affinity enrichment starting with 1 mg protein. For serial enrichment use the flowthrough and enrich with ¼ of the beads from the other PTM Scan Tube for affinity enrichment of the second modification. For the one pot enrichment (succinyl and acetyl) from 1 mg of starting material (protein lysate) use ¼ of the beads from one acetyl PTM Scan Tube plus ¼ of the beads from one succinyl PTM Scan Tube.
5. Pipette re-suspended peptides directly onto washed CST beads.
6. Place in cold room overnight on rotator or mixer.

**Day 3 – Elution and Stage tip clean-up (1-3 hours):**

**Materials**

- IAP Buffer
- 0.15% TFA
- Corning 200 µL flat-end gel loading tips
- Solid Phase Extraction Disk
- Acetonitrile
- 50% ACN, 49.8% water, 0.2% FA
- 0.2% FA in Water
- Peptide standard

1. Spin 30 seconds in cold room at 2,000 rcf.
2. Remove peptide solution and save.
3. Add 1 mL cold IAP buffer to beads to wash. Mix by inverting 5 times. Spin 30 seconds in cold room at 2,000 rcf. Remove IAP wash with aspirator.
4. Repeat step 10 once for a total of **two IAP washes.**
5. Add 1 mL cold B+J water to beads. Mix by inverting 5 times. Spin 30 seconds in cold room at 2,000 rcf. Remove water wash with aspirator.
6. Repeat step 12 twice for a **total of 3 water washes.**
7. **After the last water wash, spin once more in the cold room 30 seconds at 2,000 rcf to collect any remaining water from the sides of the Eppendorf tube. Aspirate any remaining water with gel loader tip.**
8. Add 55 µL 0.15% TFA in B+J water to beads. Incubate at room temp for 10 minutes. Tap bottom of the tube to mix intermittently.
9. Spin beads 30 seconds in mini microfuge at room temp. Remove eluted peptides with flat-tipped gel loader tip and save.
10. Add 45 µL 0.15% TFA in B+J water to beads. Incubate at room temp for 10 minutes while intermittently tapping bottom of tube to mix.
11. Spin beads 30 seconds in mini microfuge at room temp. Remove second elution by putting a **flat-ended gel loader tip at the bottom of the beads** and combine with the first elution.
12. Spin eluted peptides at 12,000 rcf for 5 minutes at room temp to pellet any beads that carried over.

**Stage Tip cleanup, ~2-5hr (1hr procedure + speedvac time):**

****Spin ~1 minute at 3,000 rcf to force liquid through at each step

1. Make a StageTip for each sample:
   1. Press the tip of an 18 gauge blunt end needle against c18 disk and twist to cut out a small circle of C18 from the disk, which should now be in the needle tip.
   2. Repeat A once so that two pieces of C18 are in the needle tip
   3. Insert needle containing C18 into a 200uL pipette tip
   4. Using thin LC tubing tubing (or something with a small enough diameter to fit into the needle) push the C18 out of the needle and into the pipette tip. It should be packed snugly in the tip and needs to remain in place for the next steps.
2. Wet StageTip with 100 µL of 100% ACN.
3. Wash StageTip with 100 µL 50% ACN, 49.8% water, 0.2% FA.
4. Equilibrate Stage tip with 100 µL 0.2% FA in water.
5. Repeat step 22 once for a total of two 0.2% FA equilibrations.
6. Load peptides eluted from IP.
7. Wash peptides with 100 µL 0.2% FA in water.
8. Repeat step 25 once for a total of two washes.
9. In a new tube, elute with 50 µL of 50% ACN, 49.8% Water, 0.2% FA.
10. Dry peptides completely in speedvac.
11. Resuspend peptides with your favorite injection solution + 0.1 µL HRM standards per injection (e.g. if desired resuspension volume is 10 µL, and 3 µL will be injected on column, add 0.33 uL HRM standards and 9.67 uL resuspension solution). **For single-injection SWATH workflow, add 6.8 uL 3% ACN/0.2%FA + 0.2 uL HRM standards.** Sonicate in water bath 5 minutes. Vortex at least 5 minutes. Spin at >12,000 rcf for >2 minutes and transfer to autosampler vial.
