# Supplementary material for: Simultaneous Quantification of the Acetylome and Succinylome by ‘One‐Pot’ Affinity Enrichment
Source: Proteomics. 2018 Aug 19;18(17):1800123. doi: 10.1002/pmic.201800123 (PMC6175148; doi:10.1002/pmic.201800123)
Supplement: Supplementary file 2 — Supporting information. [file PMIC-18-na-s002.docx]

**Supporting Information Fig. 1: Workflow for serial enrichment of PTM’s.** Immunoaffinity enrichments are performed with commercially available antibodies to acetylated or succinylated peptides, and each requires peptide digests from 1mg of mitochondrial protein isolated from tissue. For serial enrichments, we first enrich for one PTM, and the flow through was then collected and enriched for the other PTM. For example, if immunoaffinity enrichment of acetylated peptides was performed first, the flow through from the pulldown was collected and subsequently enriched for succinylated peptides. Enrichments were performed with both acetylation and succinylation as the primary and secondary pulldowns.

**Supporting Information Fig. 2: Peptide coefficients of variation in one-pot, acetyl-lysine, and succinyl-lysine enrichments.** A) Distribution of peptide coefficients of variation in one-pot, succinyl-lysine, and acetyl-lysine enrichments. B) Boxplot of coefficients of variation of acetylated or succinylated peptides in common between single-PTM enrichments and one-pot enrichments. Abbreviations: Ac = acetyl-lysine pulldown sites, Ac_onepot_ = acetylation sites from one-pot pulldown, Su = succinyl-lysine pulldown sites, Su_onepot_ = succinylation sites from one-pot pulldown.

**Supporting Information Fig. 3: One-pot acetylation and succinylation site-level correlations.** A) Correlation of acetylation site-level quantification of two one-pot pulldowns performed in parallel with two individual pulldowns of acetyl-lysine only. B) Correlation of succinylation site-level quantification one-pot enrichments to succinylation-only pulldowns. Abbreviations: Ac = acetyl-lysine pulldown sites, Ac_onepot_ = acetylation sites from one-pot pulldown, Su = succinyl-lysine pulldown sites, Su_onepot_ = succinylation sites from one-pot pulldown.

**Supporting Information Fig. 4: One-pot acetylation and succinylation peptide-level correlations.** A) Correlation of acetylation peptide-level quantification of two one-pot pulldowns performed in parallel with two individual pulldowns of acetyl-lysine only. B) Correlation of succinylation peptide-level quantification one-pot enrichments to succinylation-only pulldowns. Abbreviations: Ac = acetyl-lysine pulldown sites, Ac_onepot_ = acetylation sites from one-pot pulldown, Su = succinyl-lysine pulldown sites, Su_onepot_ = succinylation sites from one-pot pulldown.

**Supporting Information Fig. 5: One-pot acetylation and succinylation fragment-level correlations.** A) Correlation of acetylation fragment-level quantification of two one-pot pulldowns performed in parallel with two individual pulldowns of acetyl-lysine only. B) Correlation of succinylation fragment-level quantification one-pot enrichments to succinylation-only pulldowns. Abbreviations: Ac = acetyl-lysine pulldown sites, Ac_onepot_ = acetylation sites from one-pot pulldown, Su = succinyl-lysine pulldown sites, Su_onepot_ = succinylation sites from one-pot pulldown.

**Supporting Information Fig. 6: Serial enrichment acetylation and succinylation site-level correlations.** A) Correlation of acetylation site-level quantification of two serial enrichment pulldowns performed in parallel with two acetylation-only pulldowns. B) Correlation of succinylation site-level quantification serial enrichment enrichments to succinylation-only pulldowns. Abbreviations: Ac = acetyl-lysine pulldown sites, Ac_ser_ = acetylation sites from serial enrichment, Su = succinyl-lysine pulldown sites, Su_ser_ = succinylation sites from serial enrichment.

**Supporting Information Fig. 7: Serial enrichment acetylation and succinylation peptide-level correlations.** A) Correlation of acetylation peptide-level quantification of two serial enrichment pulldowns performed in parallel with two acetylation-only pulldowns. B) Correlation of succinylation peptide-level quantification serial enrichment enrichments to succinylation-only pulldowns. Abbreviations: Ac = acetyl-lysine pulldown sites, Ac_ser_ = acetylation sites from serial enrichment, Su = succinyl-lysine pulldown sites, Su_ser_ = succinylation sites from serial enrichment.

**Supporting Information Fig. 8: Serial enrichment acetylation and succinylation fragment-level correlations.** A) Correlation of fragment-level quantification of acetylated peptides from two serial enrichment pulldowns performed in parallel with two acetylation-only pulldowns. B) Correlation of succinylated peptide fragment-level quantification from serial enrichments to single antibody pulldowns. Abbreviations: Ac = acetyl-lysine pulldown sites, Ac_ser_ = acetylation sites from serial enrichment, Su = succinyl-lysine pulldown sites, Su_ser_ = succinylation sites from serial enrichment.

**Supporting Information Fig. 9: Database searching for acetylation and succinylation together versus individually.** Venn diagrams depicting the overlap of A) acetylated peptides and B) succinylated peptides identified from a one-pot enrichment after database searching for the acetylation PTM only (left) or both acetylation and succinylation (right).

**Supporting Information Fig. 10: Reticulocalbin-2 peptide containing both acetylation and succinylation.** A) MS2 spectrum of the acetylated (K_158_) and succinylated (K_156_) peptide QLHLK_su_DK_ac_K++. B) Table highlighting the b and y ions observed for this peptide. C) Bar graph showing the precursor and fragment intensities of this peptide following acetyl-lysine, succinyl-lysine, and one-pot pulldowns. D) Chromatograms showing the precursor and fragment peaks of this peptide following acetyl-lysine (left), succinyl-lysine (middle), and one-pot (right) enrichments. The peptide spectrum was taken from a DDA spectral library.

**Supporting Information Fig. 11: Malate dehydrogenase peptide containing both acetylation and succinylation.** MS2 spectra of the peptide, ASIKKGEDFVK, A) succinylated on K_128_, B) succinylated on K_129_, C) succinylated on K_128_ and acetylated on K_129_, and C) acetylated on K_128_ and succinylated on K_129_. Peptide spectra were taken from a DDA spectral library.

**Supporting Information Fig. 12: Differentiating fragment ions in malate dehydrogenase precursors with identical m/z .** MS2 spectra of the peptide, ASIKKGEDFVK, A) succinylated on K_128_, B) succinylated on K_129_. The extracted ion chromatograms for both precursors displays two peaks with a common set of fragments. The site-localized differentiating product ion (y7) can be used to distinguish the chromatographic peaks from each precursor.
