# Supplementary material for: Simultaneous Quantification of the Acetylome and Succinylome by ‘One‐Pot’ Affinity Enrichment
Source: Proteomics. 2018 Aug 19;18(17):1800123. doi: 10.1002/pmic.201800123 (PMC6175148; doi:10.1002/pmic.201800123)
Supplement: Supplementary file 3 — Supporting information. [file PMIC-18-na-s003.pptx]

## Slide 1
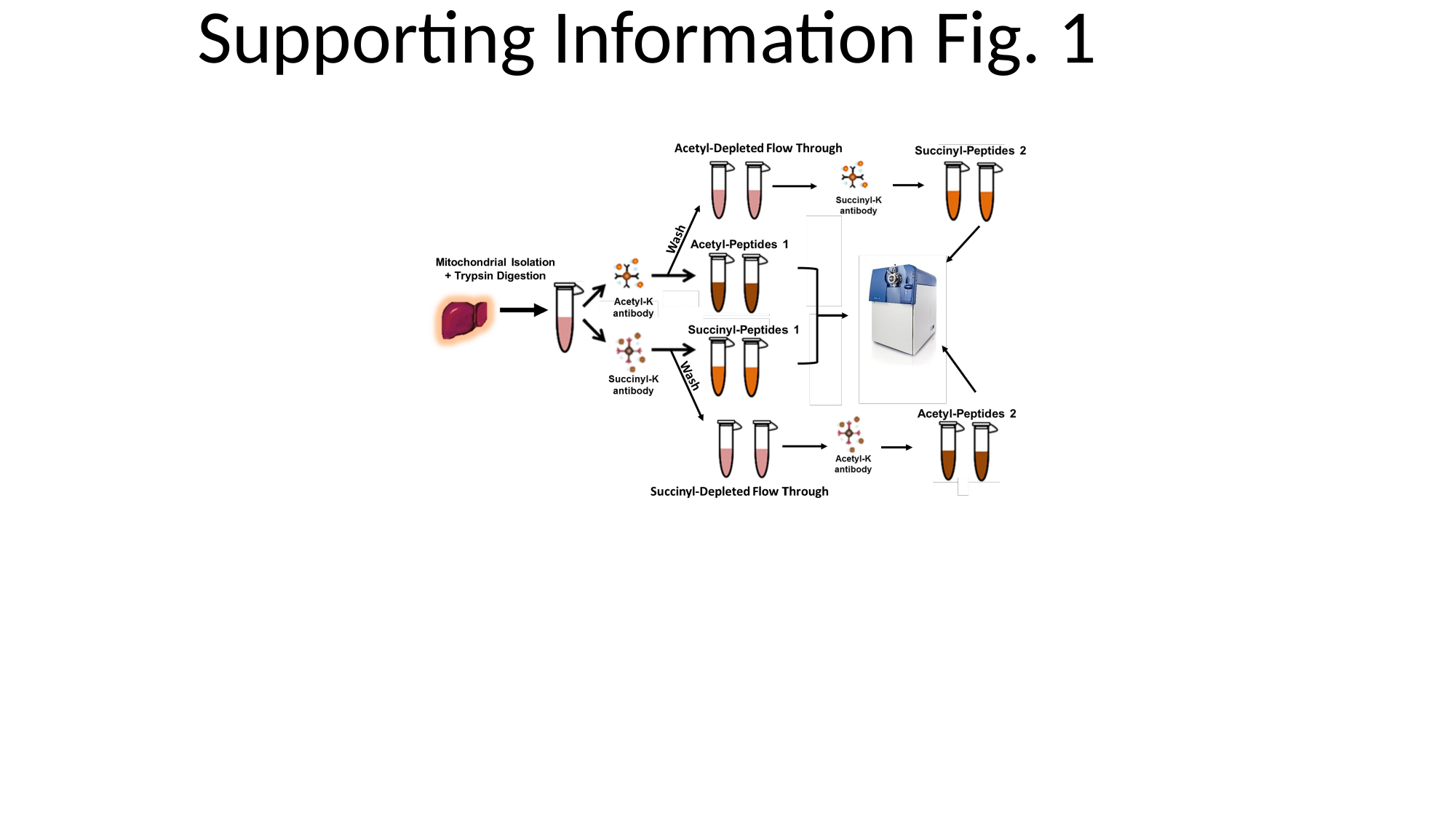

Supporting Information Fig. 1

## Slide 2
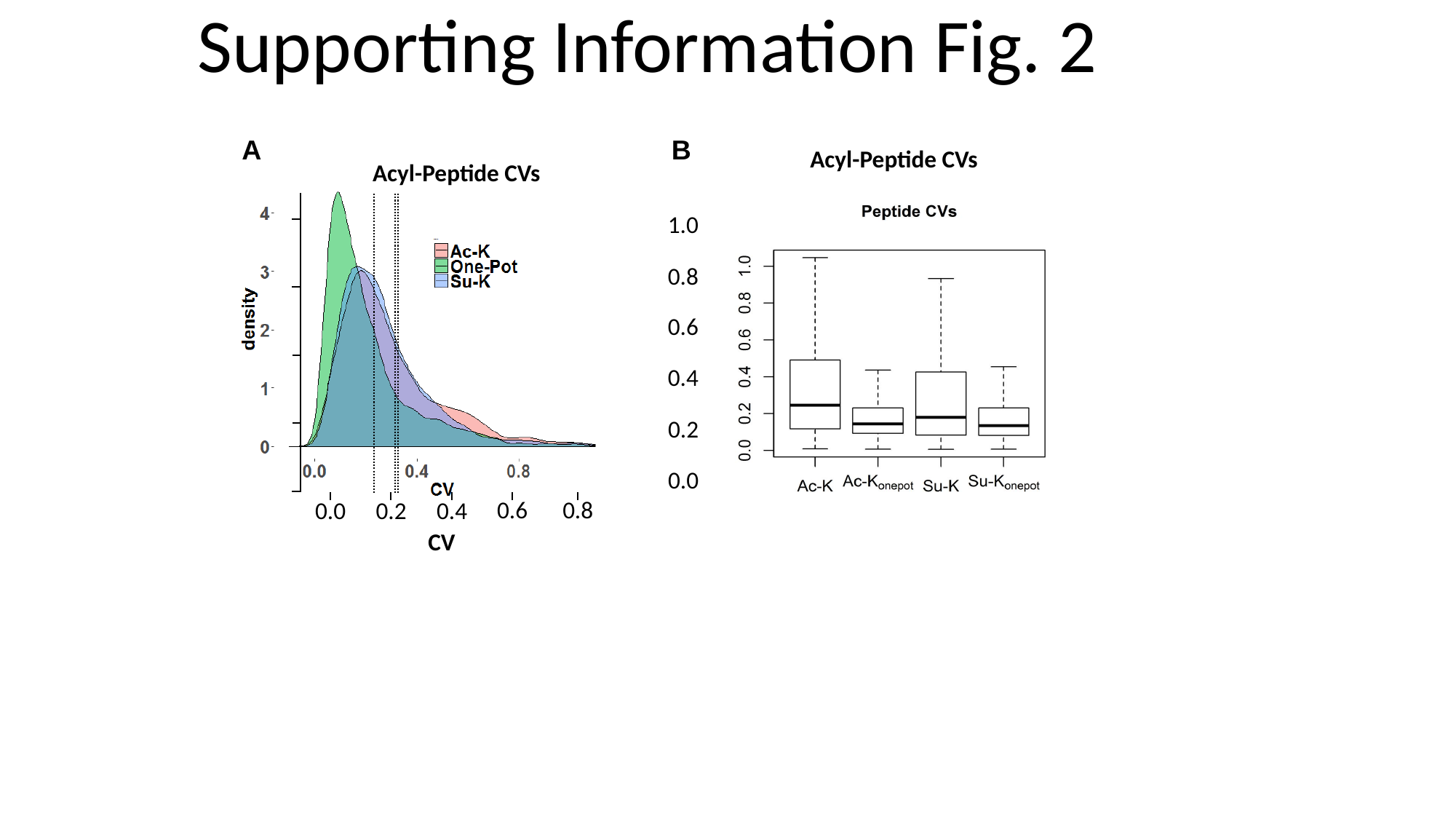

Supporting Information Fig. 2
A
B
Acyl-Peptide CVs
Acyl-Peptide CVs
CV
1.0
0.8
0.6
0.4
0.2
0.0
0.6
0.8
0.0
0.2
0.4

## Slide 3
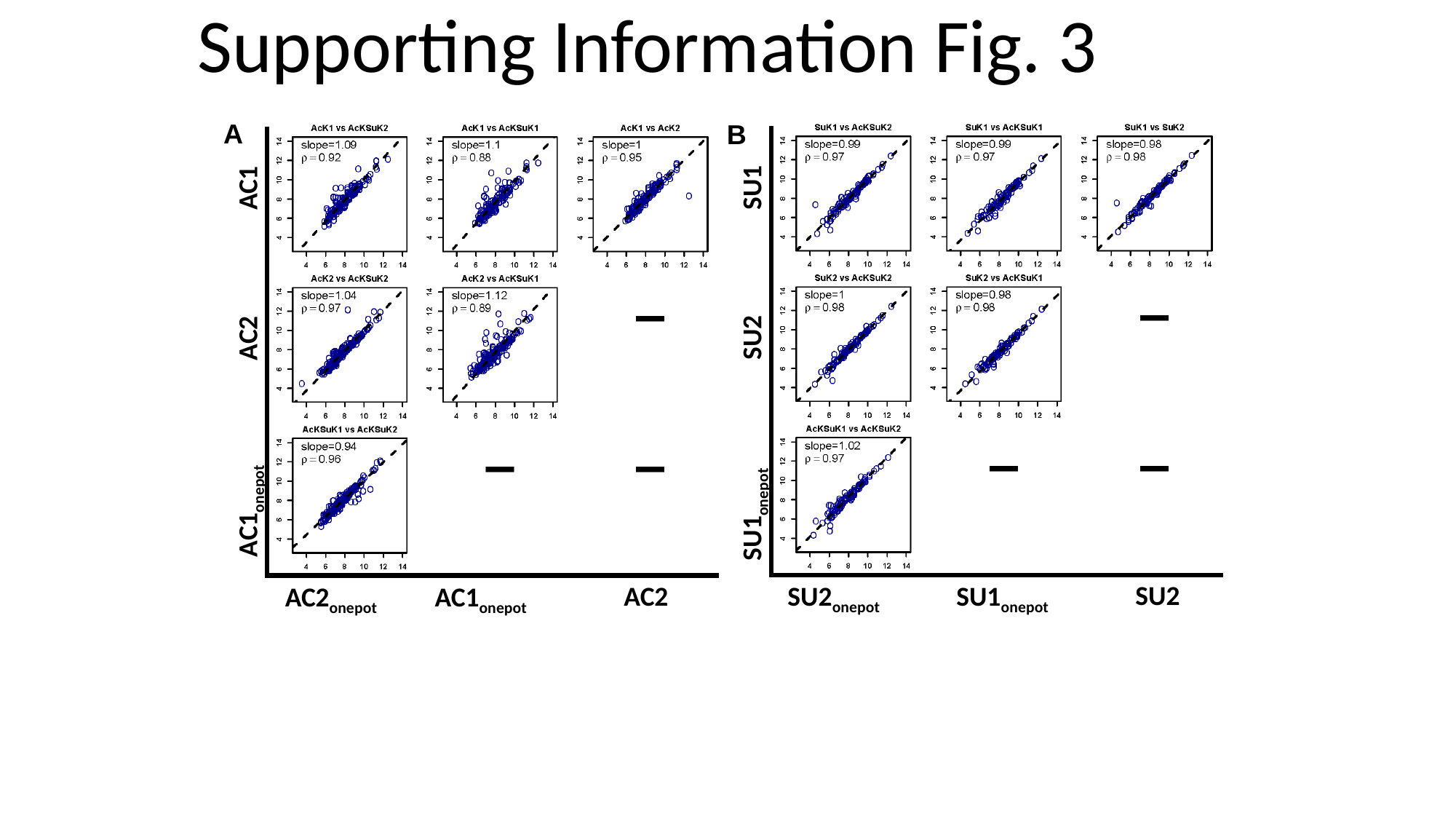

Supporting Information Fig. 3
A
B
SU1
SU2
SU1onepot
SU2
SU2onepot
SU1onepot
AC1
AC2
AC1onepot
AC2
AC2onepot
AC1onepot

## Slide 4
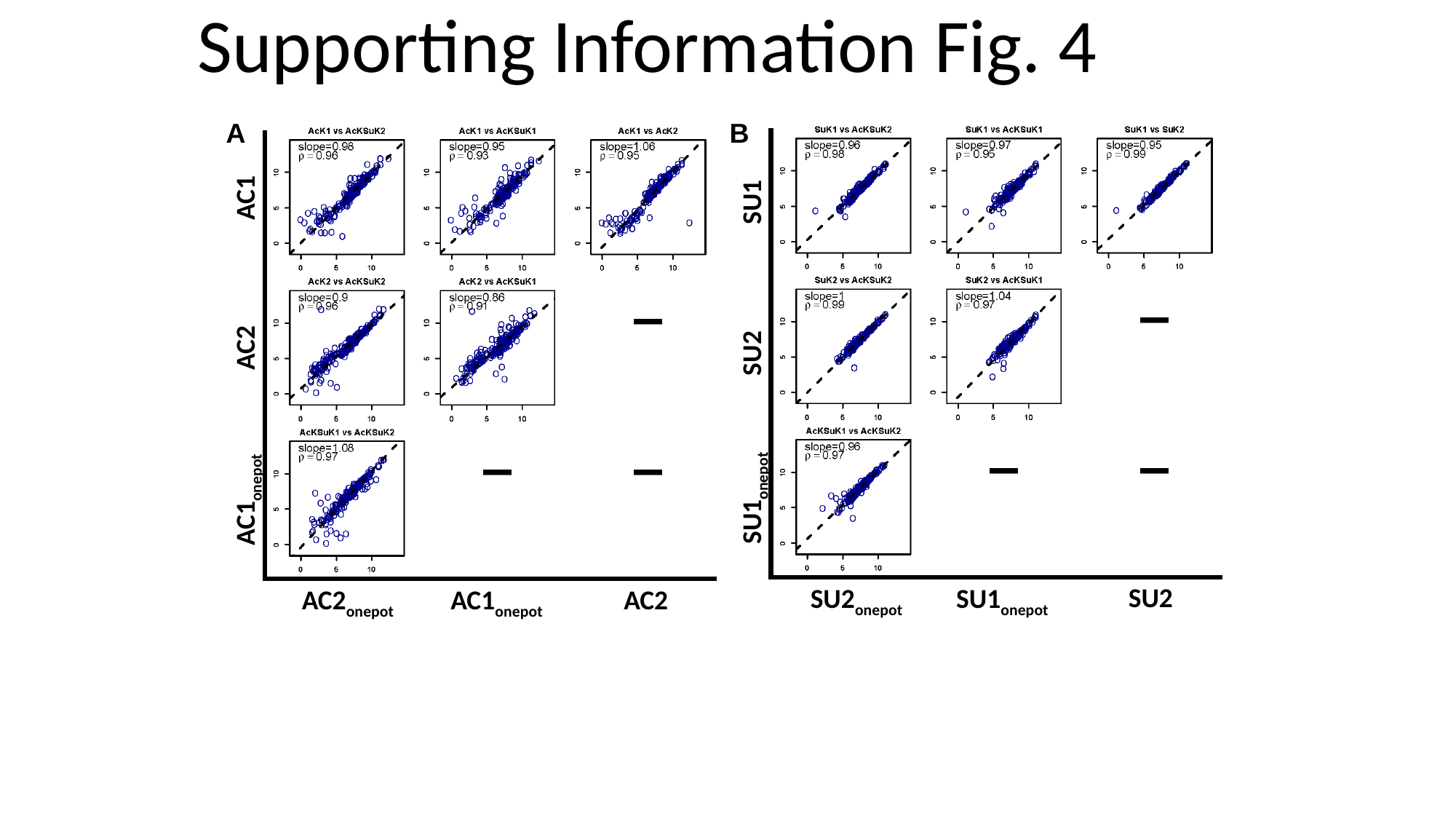

Supporting Information Fig. 4
A
B
SU1
SU2
SU1onepot
SU2
SU2onepot
SU1onepot
AC1
AC2
AC1onepot
AC2
AC2onepot
AC1onepot

## Slide 5
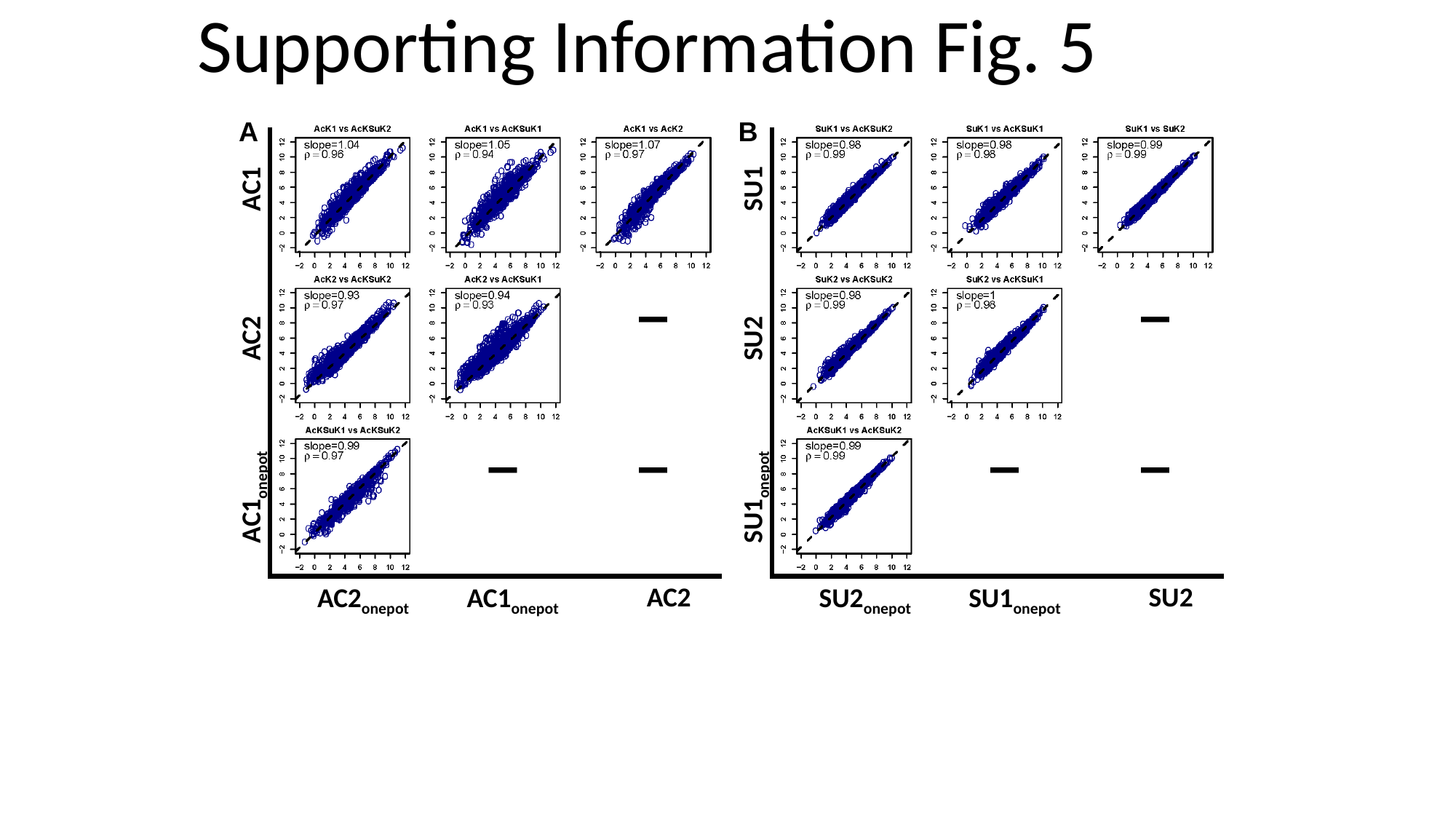

Supporting Information Fig. 5
A
B
AC1
AC2
AC1onepot
AC2
AC2onepot
AC1onepot
SU1
SU2
SU1onepot
SU2
SU2onepot
SU1onepot

## Slide 6
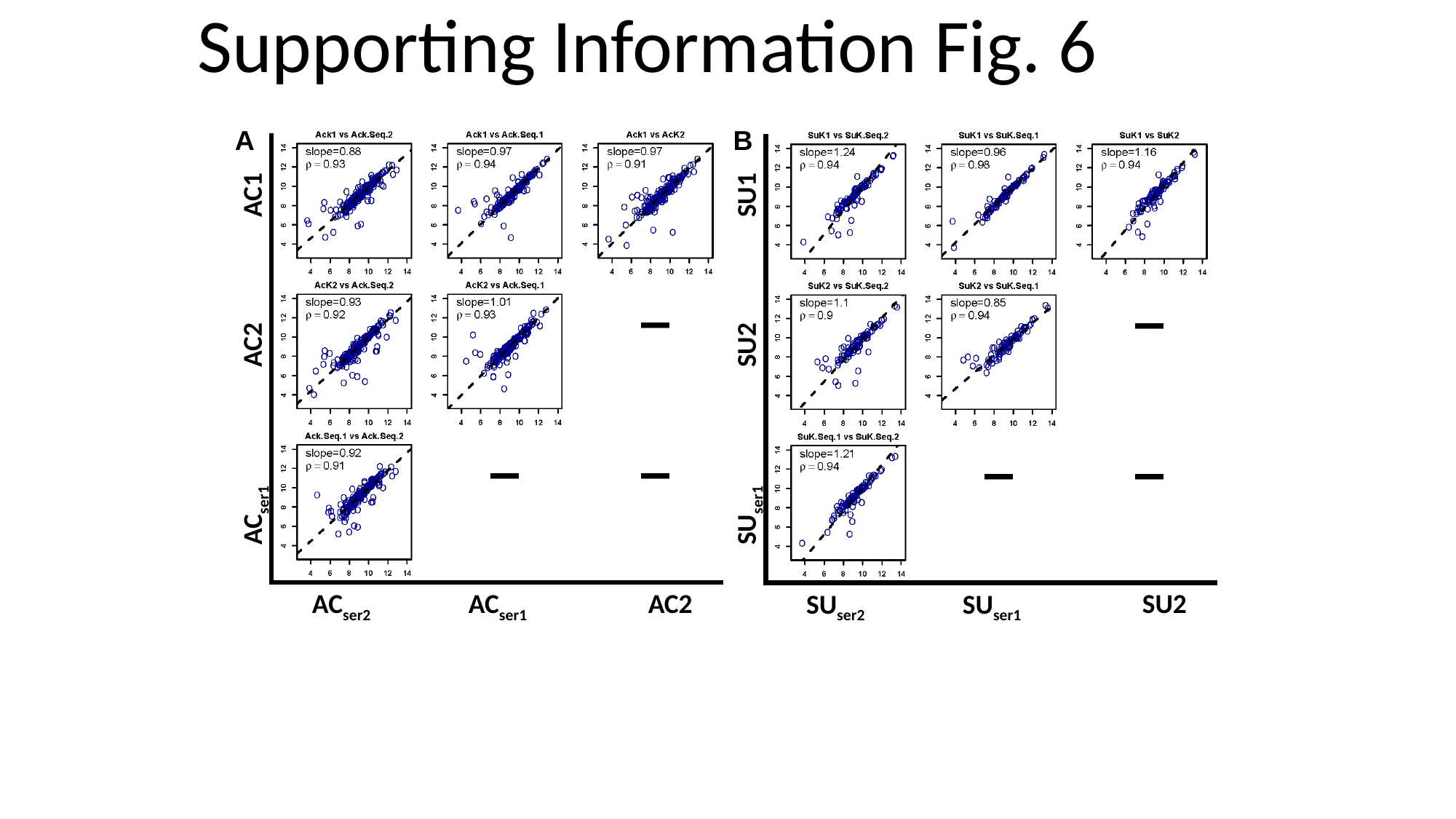

Supporting Information Fig. 6
A
B
AC1
AC2
ACser1
AC2
ACser2
ACser1
SU1
SU2
SUser1
SU2
SUser2
SUser1

## Slide 7
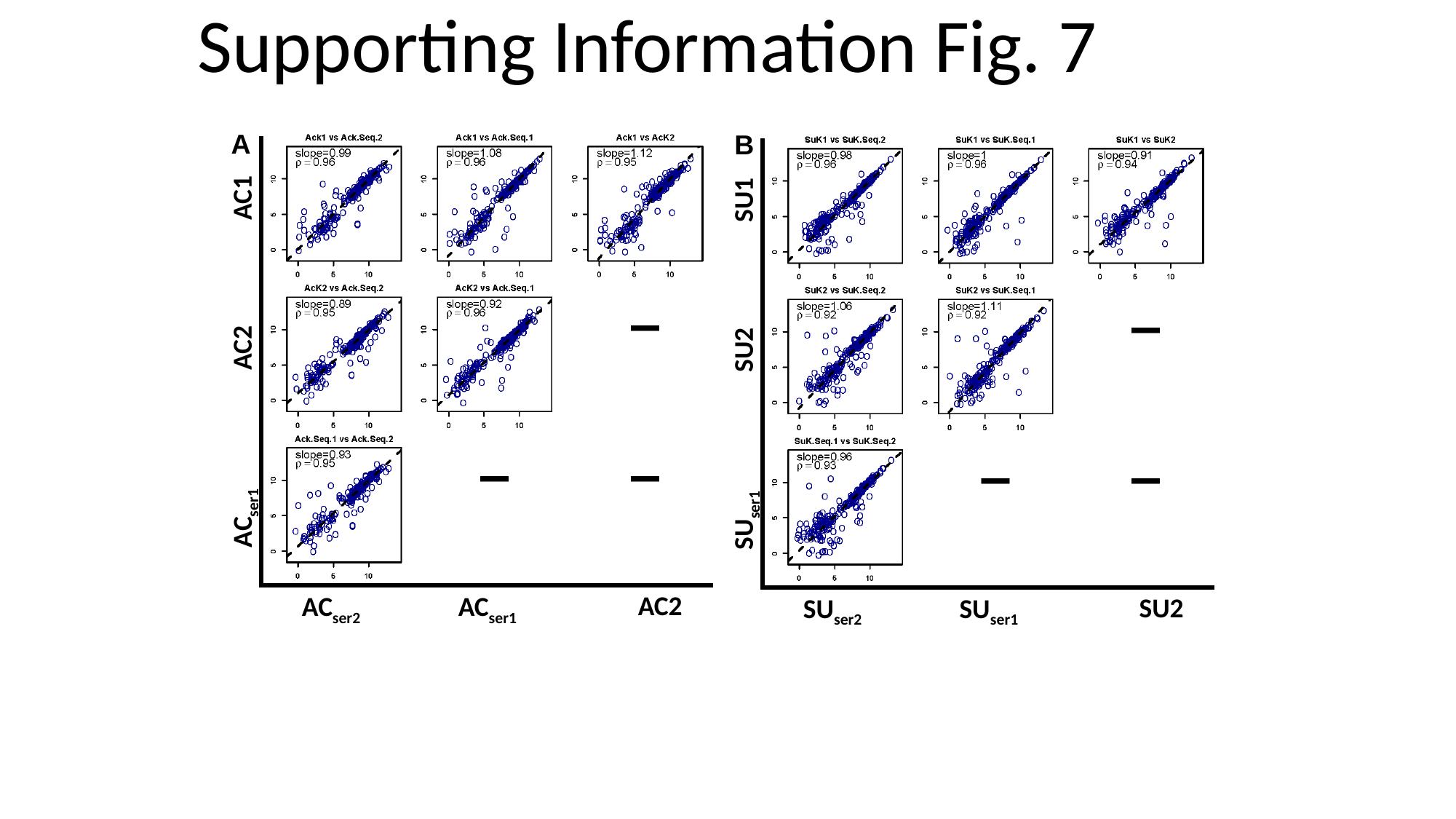

Supporting Information Fig. 7
A
B
AC1
AC2
ACser1
AC2
ACser2
ACser1
SU1
SU2
SUser1
SU2
SUser2
SUser1

## Slide 8
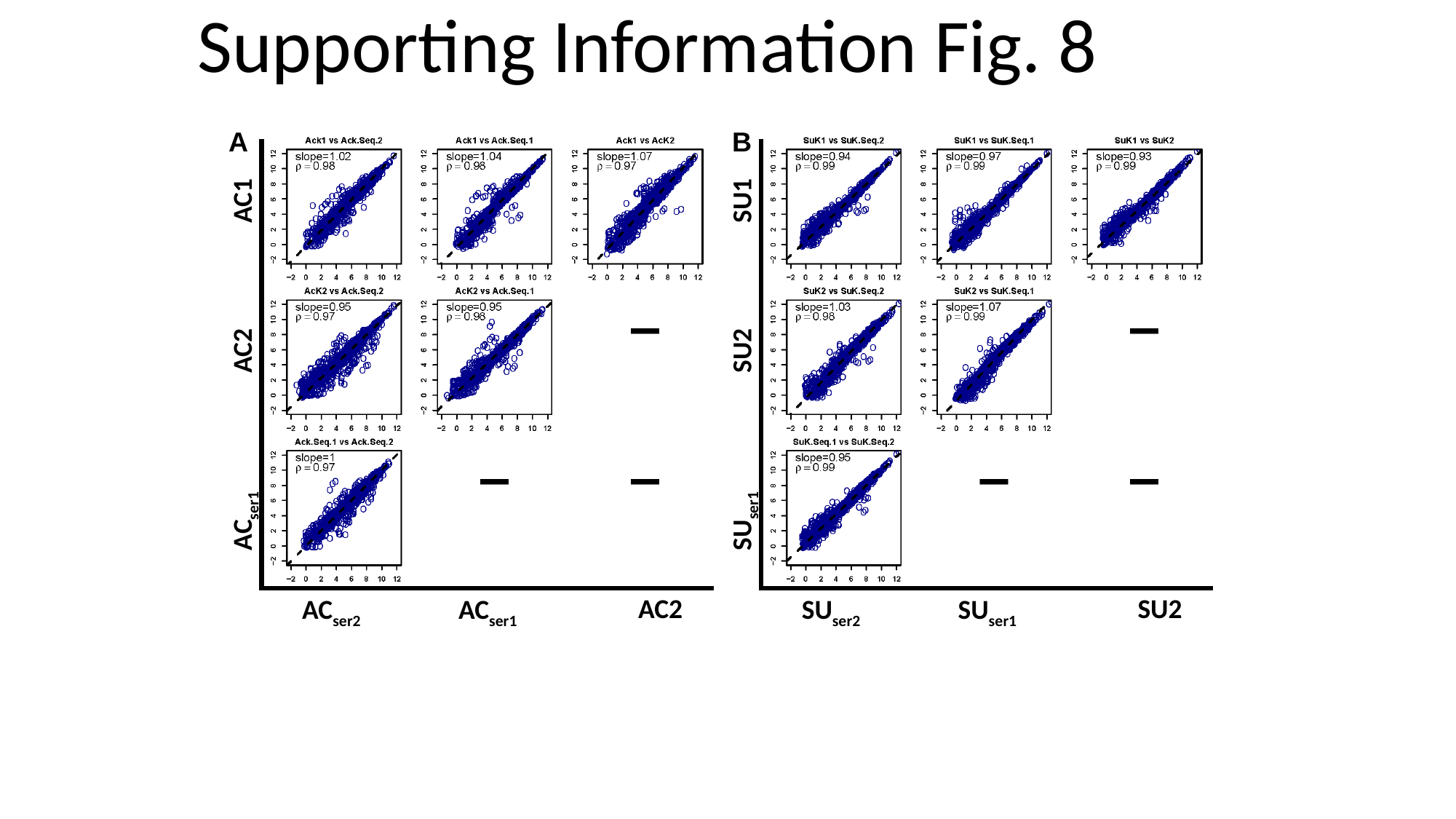

Supporting Information Fig. 8
A
B
AC1
AC2
ACser1
AC2
ACser2
ACser1
SU1
SU2
SUser1
SU2
SUser2
SUser1

## Slide 9
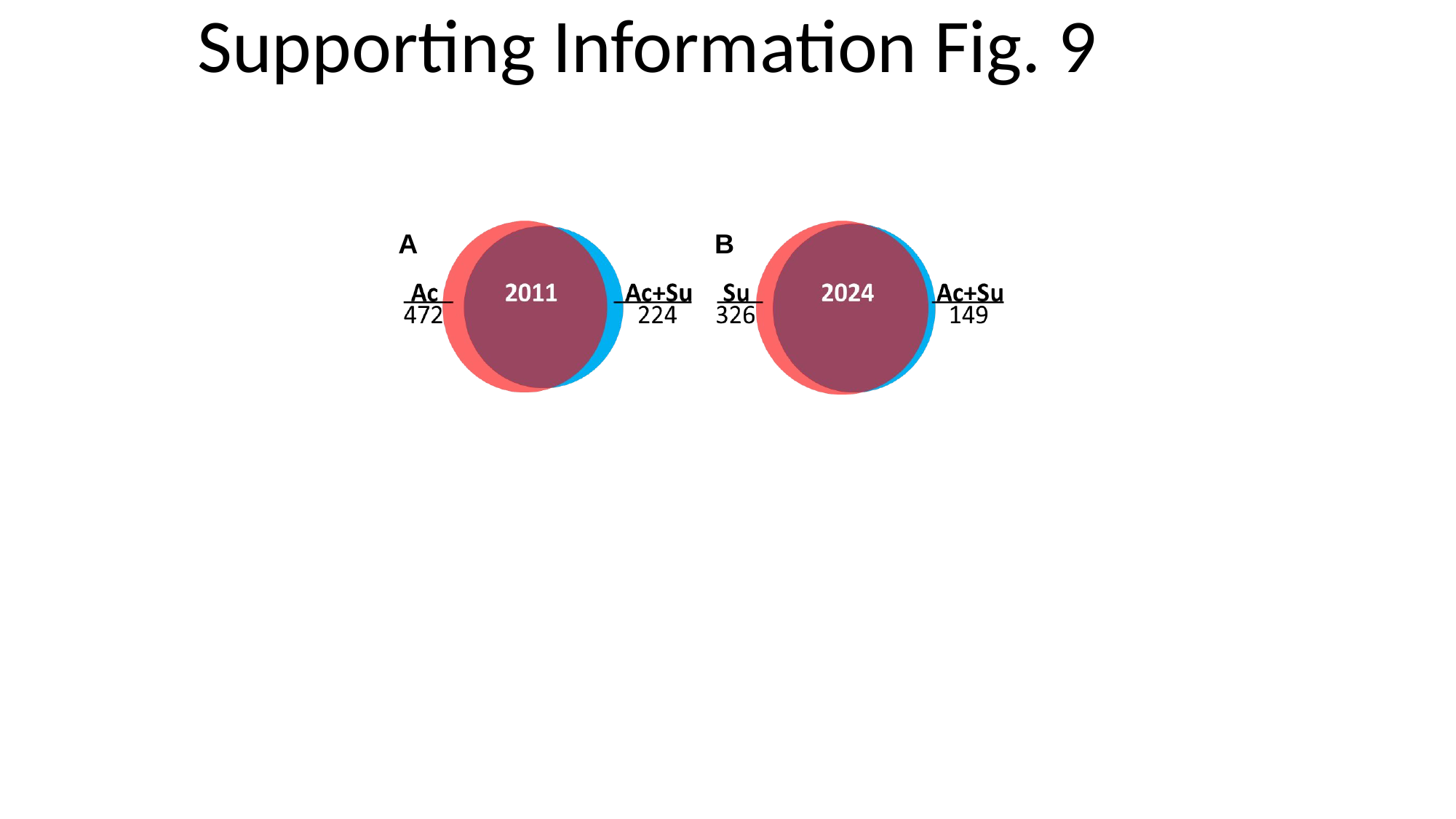

Supporting Information Fig. 9
A
B

## Slide 10
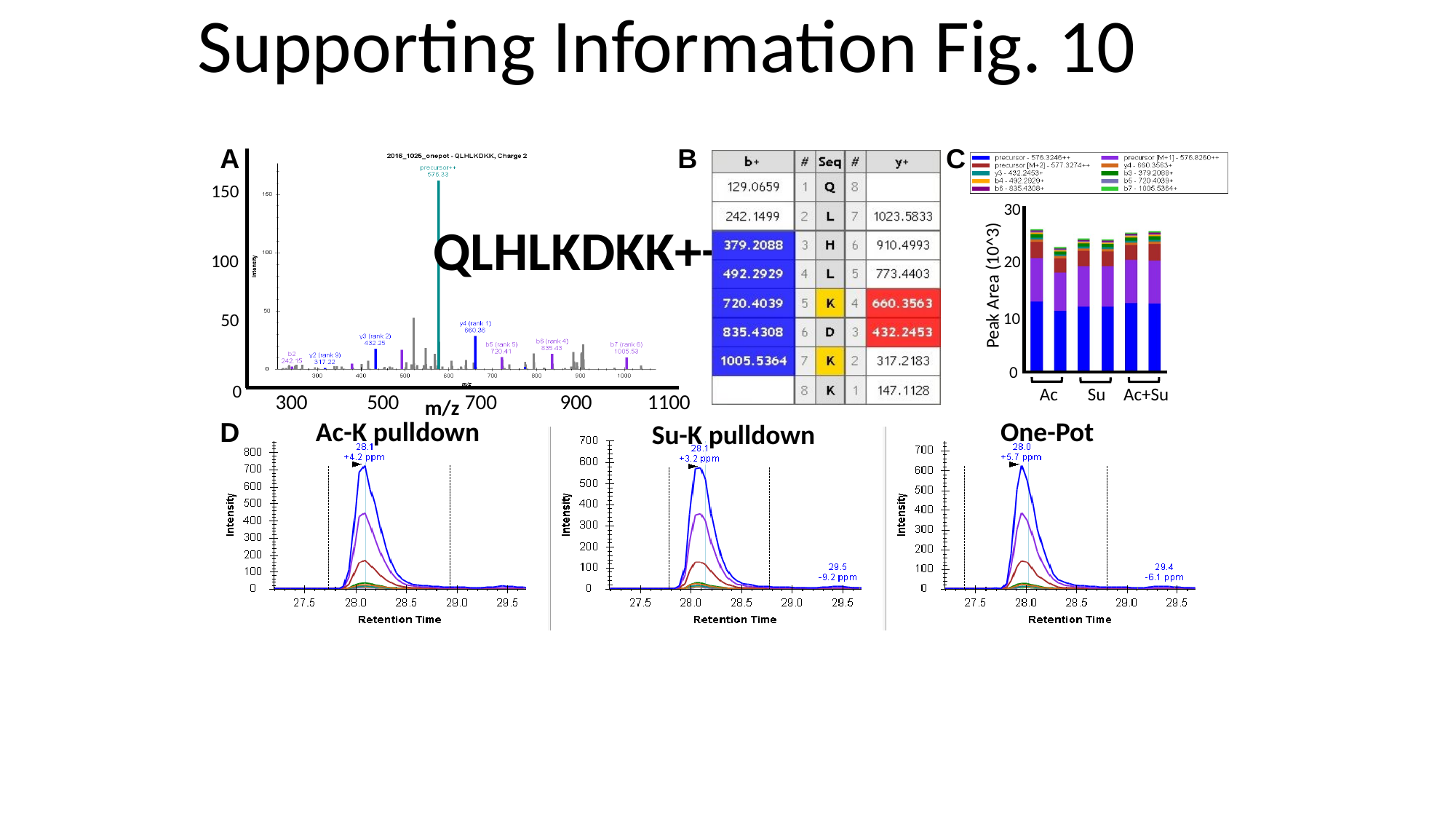

Supporting Information Fig. 10
A
C
B
150
QLHLKDKK++
100
50
0
700
900
1100
500
300
m/z
30
20
Peak Area (10^3)
10
0
Su
Ac+Su
Ac
Ac-K pulldown
One-Pot
Su-K pulldown
D

## Slide 11
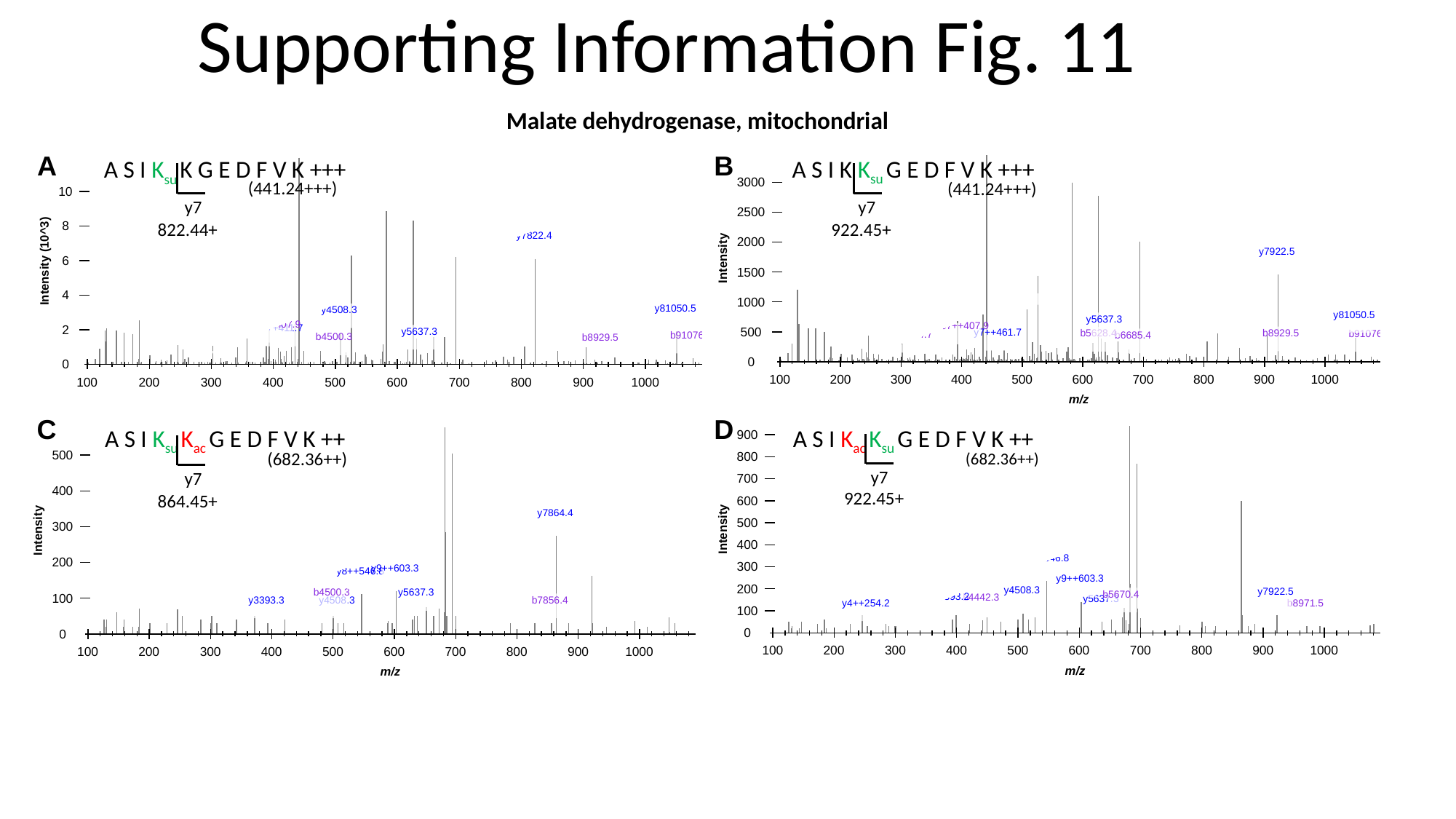

Supporting Information Fig. 11
Malate dehydrogenase, mitochondrial
A
B
A S I K Ksu G E D F V K +++
A S I Ksu K G E D F V K +++
(441.24+++)
(441.24+++)
y7
y7
922.45+
822.44+
C
D
A S I Ksu Kac G E D F V K ++
A S I Kac Ksu G E D F V K ++
(682.36++)
(682.36++)
y7
y7
922.45+
864.45+

## Slide 12
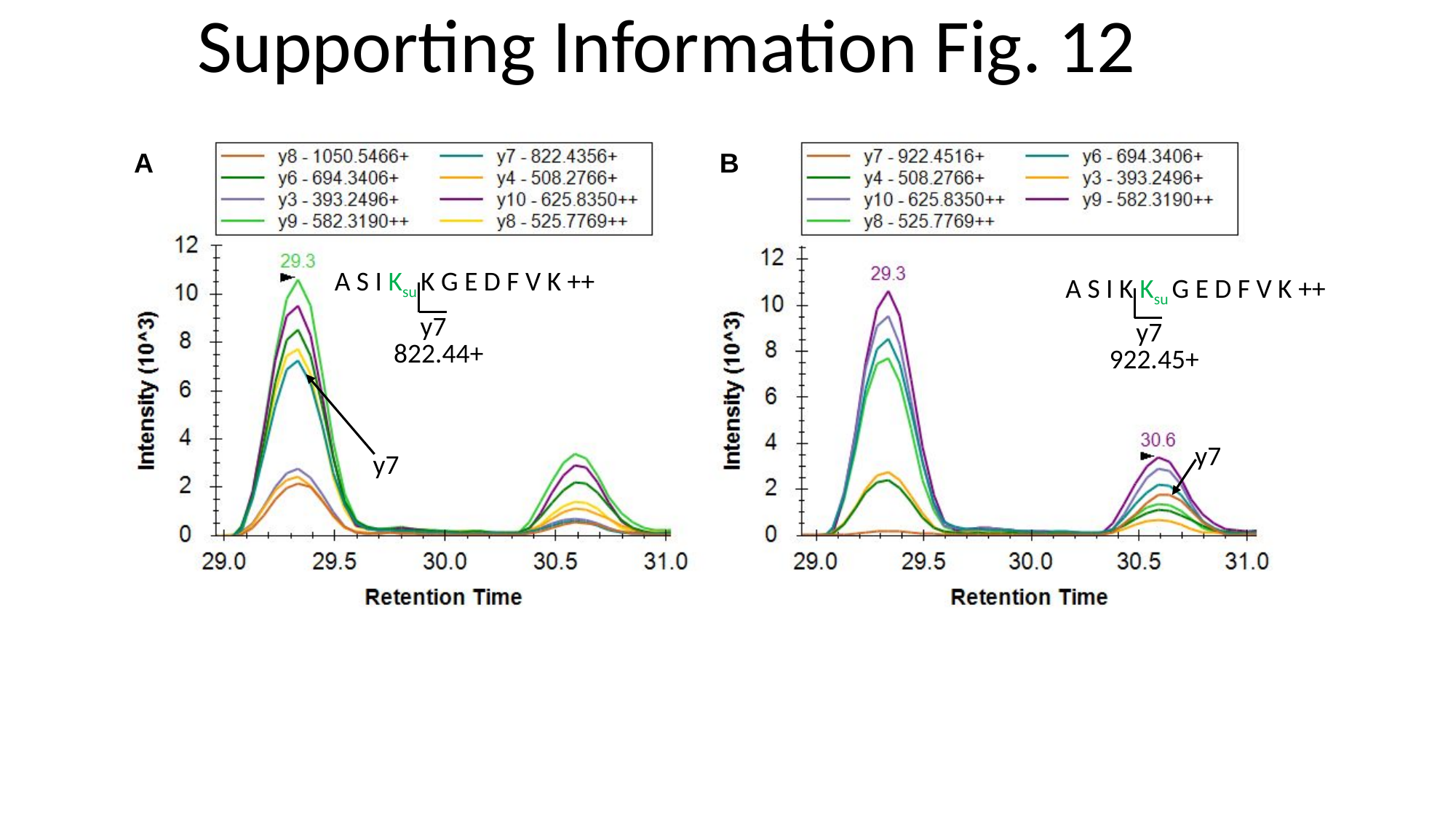

Supporting Information Fig. 12
A
B
A S I Ksu K G E D F V K ++
A S I K Ksu G E D F V K ++
y7
y7
822.44+
922.45+
y7
y7
