# Supplementary material for: Simultaneous Quantification of the Acetylome and Succinylome by ‘One‐Pot’ Affinity Enrichment
Source: Proteomics. 2018 Aug 19;18(17):1800123. doi: 10.1002/pmic.201800123 (PMC6175148; doi:10.1002/pmic.201800123)
Supplement: Supplementary file 4 — Supporting information. [file PMIC-18-na-s004.docx]

**supporting information methods**

**Materials.** Water (#AH365-4) and acetonitrile (#015-4) were obtained from Honeywell Burdick and Jackson (Muskegon, MI, USA). Triethylammonium bicarbonate buffer (#T7408), formic acid (#94318-50ML-F), nicotinamide (#N3376), trichostatin (#T8552), iodoacetamide (#I6125), and dithiothreitol (#D0632) were purchased from Sigma Aldrich (St. Louis, MO, USA). Trypsin (#V5113) was purchased from Promega (Madison, WI). Urea (#29700) and BCA protein assay kit (#23225) were purchased from Thermo Fisher Scientific (Waltham, MA). Oasis HLB columns (#186003908) were purchased from Waters (Milford, MA). HRM calibration peptides (#Ki-3003) were purchased from Biognosys.

**Sample isolation and digestion.** Proteins were obtained from mouse tissue and prepared as previously described (M.J. Rardin et al. 2013, *Proc Natl Acad Sci*). Briefly, mitochondria were isolated from the livers of SIRT5-/- (C57BL/6) mice by differential centrifugation, protein concentrations determined by BCA assay, and 1 mg of protein of each sample were then aliquoted and brought to equal volume 8M urea in 50 mM TEAB buffer, and samples were vortexed for 10 min. Samples were then reduced with 20 mM Dithiothreitol (DTT) for 30 min at 37°C, alkylated in 40 mM iodoacetamide for 30 min at room temperature, diluted 10-fold, and digested overnight at 37°C with trypsin used at 1:50 enzyme protein. Digestion was stopped by the addition of formic acid. Samples were then desalted with Oasis HLB 10 mg Sorbent Cartridges, concentrated to near dryness, and resuspended in 1.4mL immunoaffinity purification (IAP) buffer.

**PTM Enrichment.** Immunoaffinity enrichments of modified peptides were done using the PTMScan® Acetyl-Lysine Motif [Ac-K] Kit (#13416) and PTMScan® Succinyl-Lysine Motif [Succ-K] Kit (#13764) (Cell Signaling Technology, Danvers, MA), with slight modifications to the manufacturer protocol (M.J. Rardin et al. 2013, *Proc Natl Acad Sci*). In every sample and enrichment workflow - single PTM, serial, and one-pot enrichments – a total of 1 mg of starting protein was used. For both succinyl- and acetyl-lysine enrichment, we used ¼ the quantity of antibody-bead conjugate recommended in the manufacturer protocol: 20 µL or ¼ tube antibody-bead slurry (~62.5 µg immobilized antibody). For serial enrichments, antibody-beads were pelleted following incubation with the first antibody and the supernatant (containing the peptide digest) was transferred into a new tube containing the second antibody for a subsequent pulldown. For one-pot enrichments, 1 mg peptide digests were incubated in a tube containing equal parts (~62.5 µg immobilized antibody) of both the succinyl- and acetyl-lysine antibodies.

**Data Acquisition.** Samples were analyzed by reverse-phase HPLC-ESI-MS/MS using the Eksigent Ultra Plus nana-LC 2D HPLC system (Dublin, CA) combined with a cHiPLC System, which was directly connected to a quadrupole time-of-flight SCIEX TripleTOF 5600 or a TripleTOF 6600 mass spectrometer (SCIEX, Redwood City, CA). Typically, mass resolution in precursor scans was ~35,000 (TripleTOF 5600) or 45,000 (TripleTOF 6600), while fragment ion resolution was ~15,000 in ‘high sensitivity’ product ion scan mode. After injection, peptide mixtures were transferred onto a C18 pre-column chip (200 µm x 6 mm ChromXP C18-CL chip, 3 µm, 300 Å, SCIEX) and washed at 2 µl/min for 10 min with the loading solvent (H2O/0.1% formic acid) for desalting. Subsequently, peptides were transferred to the 75 µm x 15 cm ChromXP C18-CL chip, 3 µm, 300 Å, (SCIEX), and eluted at a flow rate of 300 nL/min using a 3 or 4 hr gradient using aqueous and acetonitrile solvent buffers.

All study samples were analyzed by data-independent acquisitions (DIA), or specifically variable window SWATH acquisitions. In these SWATH acquisitions, instead of the Q1 quadrupole transmitting a narrow mass range through to the collision cell, windows of variable width (5 to 90 m/z) are passed in incremental steps over the full mass range (m/z 400-1250). The cycle time of 3.2 sec includes a 250 msec precursor ion scan followed by 45 msec accumulation time for each of the 64 SWATH segments. The variable windows were determined by the complexity of the typical MS1 ion current observed within a certain m/z range using a SCIEX ‘variable window calculator’ algorithm (i.e. more narrow windows were chosen in ‘busy’ m/z ranges, wide windows in m/z ranges with few eluting precursor ions). For the creation of a spectral library for analysis by the library-based DIA workflow, data-dependent acquisitions (DDA) were carried out on two one-pot enrichments to obtain MS/MS spectra for the 30 most abundant precursor ions (100 msec per MS/MS) following each survey MS1 scan (250 msec), yielding a total cycle time of 3.3 sec. For collision induced dissociation tandem mass spectrometry (CID-MS/MS), the mass window for precursor ion selection of the quadrupole mass analyzer was set to ± 1 m/z using the Analyst 1.7 (build 96) software.

**Data Analysis.** To build a spectral library, two raw files obtained by DDA analysis of two samples enriched by one-pot were searched using ProteinPilot software (Sciex), and search results were then imported into Spectronaut software (R. Bruderer et al., 2018, *Mol Cell Proteomics*) (Biognosys) to create a spectral library. DIA raw files were then processed and quantified in Spectronaut using this library. One set of DIA data obtained from single-PTM enrichments, serial enrichments, and one-pot enrichments was analyzed using an early development version of the open-source PIQED pipeline (J.G. Meyer et al., 2017, *Nat Meth*). (https://github.com/jgmeyerucsd/PIQEDia/), which utilizes a workflow of open-source MS tools to perform analysis of DIA raw files**.** First, mass spectrometric WIFF files were converted to mzML using the SCIEX data converter version 1.3, and subsequently mzML files were converted to mzXML files using ProteoWizard (M.C. Chambers et al, 2012, *Nat Biotech*). DIA/SWATH acquisitions were processed using the DIA-Umpire (C.C. Tsou et al., 2015, *Nat Meth*) signal extraction module, which detects correlated precursor and fragment ion features and assembles them into pseudo-tandem MS/MS spectra stored in mgf files. These pseudo MS/MS spectra derived from all SWATH acquisitions were then searched using the database search engines xTandem!, comet (J.K. Eng et al., 2012, *Mol Cell Proteomics*),and MSGF+ (S. Kim et al. 2014, *Nat Commun*). Search results were then refined with PeptideProphet (A. Keller et al., 2002, *Anal Chem*) and combined with iProphet (D. Shteynberg et al., 2011, *Mol Cell Proteomics*). For the database searches, the enzyme was set to Trypsin and fixed modifications were set as carbamidomethylation for cysteine residues. Variable modifications included methionine oxidation (+15.99), acetylation (+42.01), glutamate to pyroglutamate conversion (-17.02), and succinylation (+100.01). False discovery rates (FDR) were required to be <0.01. The search engine results were subsequently used in Skyline to build spectral libraries for SWATH data processing in Skyline (B. MacLean et al., 2010, *Bioinformatics*), an open source software project (<http://proteome.gs.washington.edu/software/skyline>). Quantitative SWATH MS2 data analysis was based on extracted ion chromatograms (XICs) of up to 10 of the most abundant fragment ions in the identified spectra. In addition, the XICs for the top 3 resulting precursor ions were also extracted. Extracted peak areas were summed per target peptide/protein. Two different and independent sets of experiments were performed, and 3 sample replicates per pulldown condition were investigated (including additional technical replicates for MS acquisition). Significance was assessed using two-tailed Student’s t-test requiring p-values<0.05. In some cases, spiked HRM peptide standards (Biognosys) were used for peptide normalization.

**Enrichment Analysis.** Enrichment analysis of indiscriminate acylation sites (sites of both acetylation and succinylation) was performed in the ConsensusPathDB tool (<http://cpdb.molgen.mpg.de/>). Enrichment of protein complex-based sets was performed with a minimum complex size of 2, minimum overlap with input data of 2, and a p-value cutoff of 0.01.

**Data Accession.** The mass spectrometric data have been deposited in the MassIVE repository (accession # MSV00081906, <ftp://massive.ucsd.edu/MSV000081906>) and ProteomeXchange (accession # PXD008640, <http://proteomecentral.proteomexchange.org/cgi/GetDataset?ID=PXD008640>). Additional supplemental materials including supplemental methods, quantitative details for acyl peptides and sites are also available at MassIVE. Spectral libraries of acylated peptides are available on PanoramaWeb (<https://panoramaweb.org/project/Schilling/OnePot_Basisty/begin.view?>).
